# Food groups and risk of chronic disease: a protocol for a systematic review and network meta-analysis of cohort studies

**DOI:** 10.1186/s13643-016-0302-9

**Published:** 2016-07-27

**Authors:** Lukas Schwingshackl, Anna Chaimani, Angela Bechthold, Khalid Iqbal, Marta Stelmach-Mardas, Georg Hoffmann, Carolina Schwedhelm, Sabrina Schlesinger, Heiner Boeing

**Affiliations:** 1 German Institute of Human Nutrition Potsdam-Rehbruecke (DIfE), Arthur-Scheunert-Allee 114-116, 14558 Nuthetal, Germany; 2 Department of Hygiene and Epidemiology University of Ioannina School of Medicine, Medical School Campus, University of Ioannina, Ioannina, 45110 Greece; 3 German Nutrition Society, Godesberger Allee 18, 53175 Bonn, Germany; 4 Department of Pediatric Gastroenterology and Metabolic Diseases, Poznan University of Medical Sciences, Szpitalna 27/33, 60-537 Poznan, Poland; 5 Department of Nutritional Sciences, University of Vienna, Althanstraße 14, UZA II, 1090 Vienna, Austria; 6 Department of Epidemiology and Biostatistics, School of Public Health, Imperial College London, St. Mary’s Campus, Norfolk Place, Paddington, London W2 1PG UK

**Keywords:** Food, Diet, Network meta-analysis, Chronic disease

## Abstract

**Background:**

There is a lack of systematic and comprehensive evaluations whether food intakes lower or increase risk of chronic diseases. In this network meta-analysis of prospective cohort studies, we aim to evaluate the effects of different foods on risk of chronic diseases.

**Methods/design:**

We will search PubMed and EMBASE. This will be supplemented by a hand search and author contacts. Citations, abstracts, and relevant papers will be screened for eligibility by two reviewers independently. Prospective cohort studies will be included if they meet the following criteria: (1) evaluate the association of single food or food groups with all-cause mortality, cardiovascular diseases (incidence and mortality), cancer (incidence and mortality) or risk of type 2 diabetes. The following food groups will be evaluated: whole grains, refined grains, vegetables, fruits, nuts, legumes, eggs, dairy products, fish, red meat, processed meat and sugar-sweetened beverages; (2) include participants ≥18 years of age; and (3) study population were free of outcome(s) of interest at the onset of the study. To assess study quality, we will extract the following characteristics: study size, duration of follow-up, dietary assessment method, assessment of outcome and adjustment factors. If the identified studies appear sufficiently similar within and across the different comparisons between pairs of food groups, we will estimate summary-relative effects using random effects network meta-analysis. Subgroup and meta-regression analyses will be performed stratified by different follow-up cut-points, geographical region and sex.

**Discussion:**

This is a presentation of the study protocol only. Results and conclusions are pending completion of this study. Our systematic review will be of great value to national and international authorities for evidence-based nutritional recommendation/guidelines, regarding the implementation of food-based dietary guidelines for prevention of chronic diseases. Moreover, our results can be implemented to develop new diet quality indices/scores.

**Systematic review registration:**

PROSPERO CRD42016037069

**Electronic supplementary material:**

The online version of this article (doi:10.1186/s13643-016-0302-9) contains supplementary material, which is available to authorized users.

## Background

Globally in 2013, the total number of deaths across all age groups rose to approximately 55 million. Seventy percent of these deaths were caused by non-communicable diseases, with cardiovascular or circulatory diseases being responsible for 32 % of the fatalities (15 % by ischemic heart disease, 12 % by stroke and approximately 3 % by type 2 diabetes) followed by cancer mortality with 15 % [1].

In nutrition research, randomized controlled trials often address short-term interventions (evaluating many dietary hypotheses) in high risk groups with intermediate outcomes. There are only a few long-term intervention studies in nutrition research and all of them evaluating many dietary hypotheses in one trial [2–4]. Therefore, in nutrition research, well-designed prospective cohort studies are the main source of evidence to address decade long exposures populations with hard clinical endpoints [5]. Large prospective cohort studies have shown that 60–75 % of coronary and 36 % of cancer incidents can be explained by lifestyle factors such as unhealthy diets, overweight, obesity, physical inactivity, smoking and excessive alcohol intake [6]. According to the Global Burden of Disease Group in 2012, unhealthy diet is the leading risk factor for death and disability [7]. A high intake of alcohol, red meat, processed meat, sugar-sweetened beverages, collectively accounted for 10 % of global disability-adjusted life years in 2010 [7]. In addition, a diet low in fruits, vegetables, milk, nuts, seeds, seafood and whole grains might also contribute to risk of death and chronic diseases.

Previous pairwise meta-analyses of cohort studies showed that certain food groups such as whole grains, vegetables and fruits were associated with reduced risk of coronary heart disease, cancer, and type 2 diabetes [8–10], whereas red meat or sugar-sweetened beverages were associated with increased risk [8–10]. To the best of our knowledge, no systematic review and meta-analysis has compared simultaneously the effects of multiple dietary factors on all-cause mortality, cardiovascular disease, cancer disease and risk of type 2 diabetes. One important question that remains to be answered is which foods are associated with the most pronounced risk reduction.

Therefore our objective is to compare the impact of different foods on the risk of all-cause mortality, cardiovascular disease, cancer and type 2 diabetes using prospective cohort studies. We also aim to obtain a useful relative ranking of the different foods with respect to the prevention of chronic disease.

## Methods and design

The review was registered in PROSPERO International Prospective Register of Systematic Reviews (http://www.crd.york.ac.uk/PROSPERO/display_record.asp?ID=CRD42016037069, identifier CRD42016037069). The present systematic review protocol was planned, conducted, and reported in adherence to standards of quality for reporting systematic review and network meta-analysis protocols [11–14] (Additional file 1).

### Eligibility criteria

Studies will be included in the meta-analysis if they meet all of the following criteria.

#### Types of studies

Only studies with a prospective cohort design (including prospective cohort studies, nested case-control studies, randomized controlled trials, case-cohort studies) that are peer-reviewed and available in full-text will be eligible for the present systematic review.

#### Types of participants

Participants aged 18 or older will be eligible. Prospective studies based on children, adolescent or pregnant women will be excluded. The sample included in final analysis had to be free of the outcome(s) (cardiovascular disease and/or, cancer, and/or type 2 diabetes) of interest at the onset of the study.

### Exposure

The impact of the following twelve food groups will be evaluated: whole grains/cereals, refined grains/cereals, vegetables, fruits, nuts, legumes, eggs, dairy products (milk, cheese, yogurt), fish, red meat, processed meat and sugar-sweetened beverages. The focus will be on these 12 food groups since most diet quality indices/score were based on these [15–17].

The assessment of dietary intake is based on multidimensional exposures and in free-living populations these measurements are often not accurate. The most commonly used techniques for assessing food and drink consumption are food frequency questionnaires, dietary record, dietary history, or 24-h recall. We will include any definition of high (e.g. third tertile, or consumers) vs. low/reference (i.e. first tertile, or non-consumers) intake categories and we intend to account for the possible impact of the different definitions in additional analyses (see the *Exploration of heterogeneity and inconsistency* section).

The use of cohort studies in this systematic review makes the evaluation of transitivity challenging since the idea of ‘joint randomizability’ is not plausible in this setting [18]. However, we will assess whether transitivity is likely to hold prior to the data synthesis following the strategy described in the section *Assessment of the transitivity assumption*.

### Geometry of the network

**Fig. 1:**
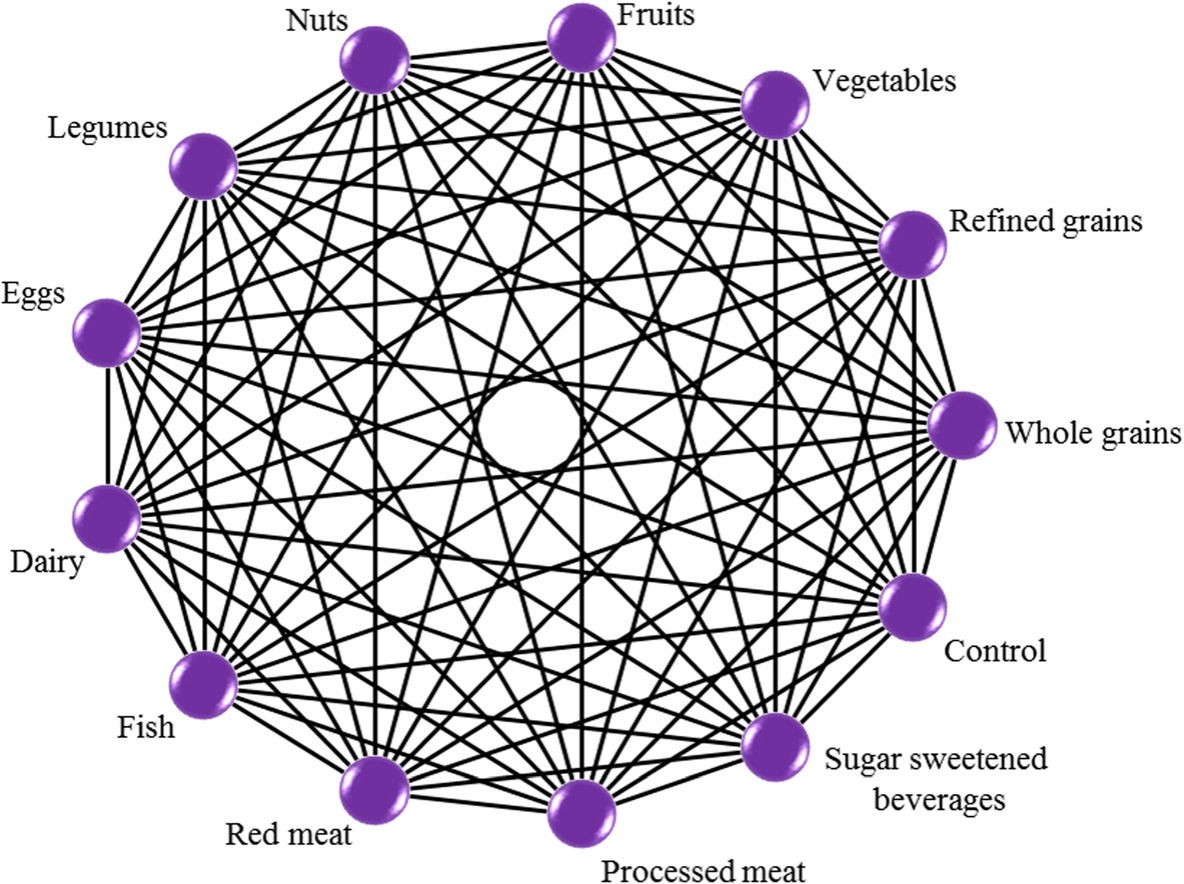
Network of all possible pairwise comparisons between the eligible dietary factors

**Fig. 2:**
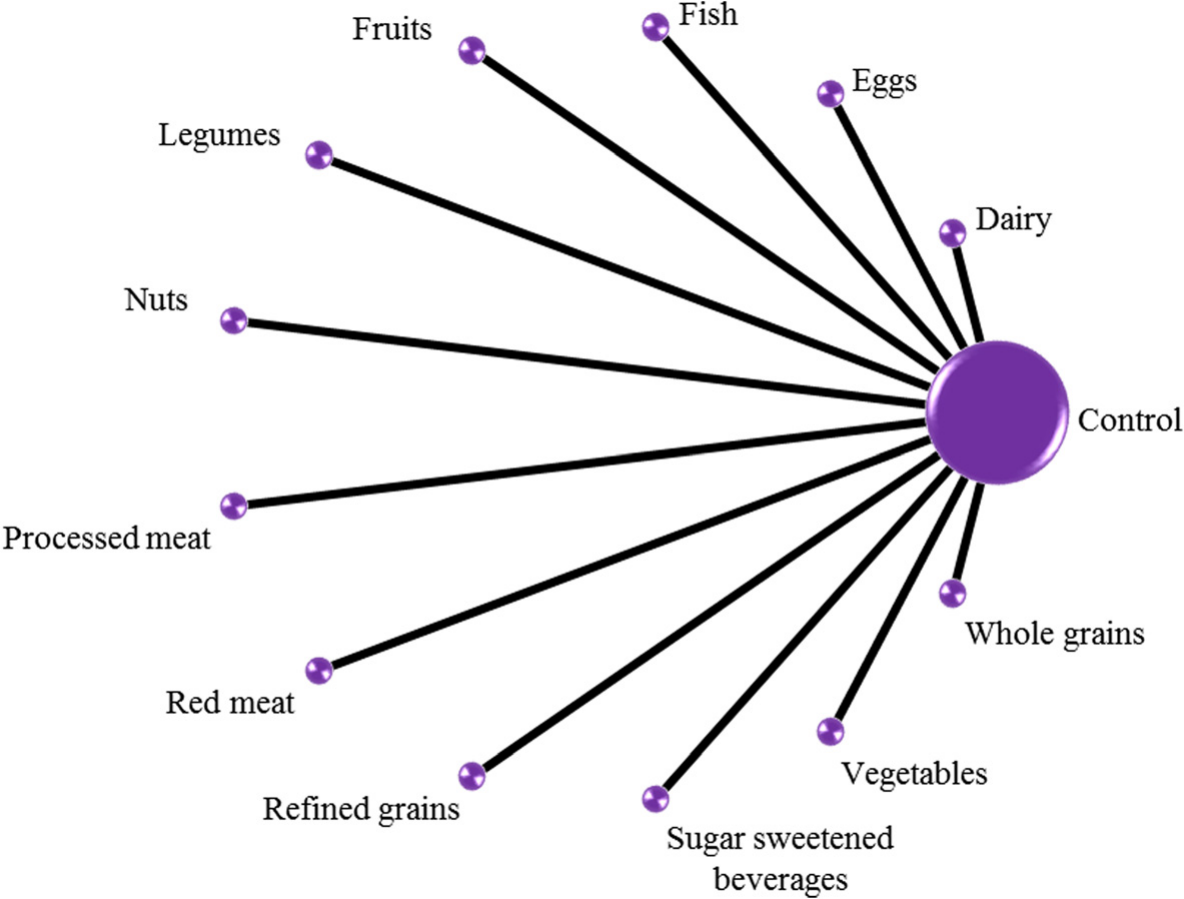
Star network of the comparisons between every eligible dietary factor and control

We do not expect to identify studies directly comparing two or more different food groups. Nevertheless, if such studies that meet the eligibility criteria will be identified, we will include them in the network. Figure 1 shows the network of all possible pairwise comparisons between the eligible dietary factors, and Fig. 2 shows a ‘star network’ between the different dietary factors and control; we expect that the structure of our final network will be close to Fig. 2.

### Outcome measures

We will consider the following outcomes:

- *Primary outcome*
  1. All-cause mortality; the following outcome assessments will be considered: death registers, record linkage, death certificates, physician records, autopsy data.
- *Secondary outcomes*
  1. Cardiovascular disease (incidence); defined accordingly: myocardial infarction, coronary heart disease, coronary artery disease (angina pectoris, myocardial infarction), stroke (haemorrhagic, ischemic), and heart failure. The following outcome assessments will be considered: record linkage (ICD codes), accepted clinical criteria, death registers, death certificates.
  2. Cancer (incidence); record linkage (ICD codes), cancer registry data, death certificates, the diagnosis of cancer should be always supported by pathological examination of tissues. We will include cancer incidence for specific cancer sides (breast, colorectal, prostate, stomach).
  3. Type 2 diabetes: the diagnosis should have been established using the standard criteria valid at the time of the beginning of the prospective study. The following outcome assessments will be considered: record linkage, National register confirmed by medical certificates, self-report of physician diagnosis, confirmed self-report of physician diagnosis, identified from register of persons receiving drug imbursement.

### Search strategy

The search strategy was developed by LS, SS and HB, and will be performed by LS, AB, MSM and KI, and differences resolved by discussion with a third reviewer (HB). We will conduct searches in PubMed (from 1966) and EMBASE (from 1980). We will search for articles of original research by using the following search terms:

1. food and beverages [MeSH Terms]
2. food*[tiab] OR whole grain*[tiab] OR refined grain*[tiab] OR cereal*[tiab] OR pasta*[tiab] OR rice*[tiab] OR potato*[tiab] OR vegetable*[tiab] OR fruit*[tiab] OR nut*[tiab] OR legume*[tiab] OR bean*[tiab] OR egg*[tiab] OR dairy[tiab] OR dairies[tiab] OR milk[tiab] OR yogurt[tiab] OR cheese[tiab] OR fish[tiab] OR seafood[tiab] OR meat[tiab] OR processed meat[tiab] OR sugar sweetened beverage*[tiab]
3. mortality OR incidence OR cardiovascular OR coronary OR stroke OR cancer OR neoplasm OR neoplastic disease OR diabetes OR vascular OR myocardial infarction
4. prospective OR follow-up OR cohort OR longitudinal
5. (#1 OR #2) AND #3 AND #4

Moreover, the reference lists from the retrieved articles; systematic reviews and meta-analyses will be checked to search for further relevant studies (umbrella review of systematic review and meta-analyses). There will be no restrictions on language or publication year. Studies published in languages other than English will be translated by international scientists in our institute.

### Study selection process

Two reviewers will independently screen titles and abstracts of all the retrieved bibliographic records. Full texts of all potentially eligible records passing the title and abstract screening level will be retrieved and examined independently by two reviewers (for each database) with the abovementioned eligibility criteria/exclusion criteria [19, 20]. Disagreements will be resolved by consensus or adjudication of another reviewer. A flow diagram will outline the study selection process and reasons for exclusions (full-text). When a study was published in duplicate, we will include the version containing the most comprehensive information (latest information in the case of follow-up studies).

### Data extraction

After determination of the study selection, two reviewers will extract independently the following characteristics: the first author’s last name, year of publication, study origin, cohort name, sample size, number of cases, age at entry, sex, study length (follow-up in years), outcome, outcome assessment, assessment of diet, quantity of diet, risk estimate (most adjusted measures) (hazard ratios (HR), risk ratios (RR) or odds ratios (OR) with their corresponding 95 % confidence intervals (CIs)) and adjustment factors using our own checklist (piloting of the tool based on three studies will be performed).

When a study provides several risk estimates, the multivariate adjusted model will be chosen. If only separate risk estimates for male and female participants will be available in one study, data will be pooled and treated as one study.

### Risk of bias assessment

To assess the risk of bias of the cohort studies, we will assess ascertainment of exposure (e.g. validated, calibrated FFQ, 24 h recall/diet history/diet records for multiple days will be judged as low risk of bias; whereas un-validated FFQ, single 24 h recall/diet records/diet history will be judged as high risk of bias studies), assessment of outcome (e.g. record linkage according ICD codes, accepted clinical criteria, independent or blind assessment will be judged as low risk of bias; whereas self-reported not validated assessment will be judged as high risk of bias studies), adequacy of follow-up depending on the outcome (e.g. stratified by the median duration of follow-up: e.g. ≥10 years for CVD will be judged as low risk of bias; whereas <10 years for CVD will be judged as high risk of bias), and adjusted basic model and outcome relevant adjustments (e.g. adjustment for the most important factors: sex, age, education, smoking and physical activity, BMI will be judged as low risk of bias studies; whereas low number of adjustments will be judged as high risk of bias), based on our own developed tool [21]. Studies will be classified as being at low risk of bias in general only if none of the domains established a high risk of bias, and at moderate overall risk of bias if they were at high risk for one domain only. In all other cases, studies were classified as being overall at high risk of bias.

### Dealing with missing data

We will try to obtain relevant missing data from authors of the included cohort studies (by e-mail). If we will not be able to obtain the missing data we will exclude the cohort from the network meta-analysis (NMA). We do not expect high missing data rate, since this phenomenon is not common in meta-analysis of observational studies [15, 18, 22].

### Evaluation of synthesis assumptions and statistical analysis

#### Description of the available data

We will generate descriptive statistics for study and population characteristics describing the available data and some important variables (e.g. age, length of follow-up, outcome relevant baseline risk factors, etc.) for each pairwise comparison. We will present the available direct comparisons between different food groups and control using a network diagram for each outcome [23]. The size of the nodes will be proportional to the sample size/number of cases exposed to each dietary factor and the thickness of the lines proportional to the inverse variance of the respective direct relative effects. We will also use the contribution matrix to identify the direct comparisons with greater influence in the network relative effects [23, 24].

#### Standard pairwise meta-analyses

We will perform three types of analysis:

1. High vs. low intake meta-analyses: summary risk will be estimated for high versus low intake of single food or food groups and risk of chronic diseases by applying random effect models.
2. Dose-response meta-analyses: we will investigate the association between intake of dietary factors as a continuous variable and risk of chronic diseases, by performing a dose-response meta-analysis as described by Orsini et al. and Greenland and Longnecker [25, 26]. This method requires for at least three exposure categories: the quantified exposure value and the RRs with the respective 95 % CI, as well as the number of cases and person-years. If studies did not report on distribution of person-years in single categories, but provided information on number of cases and total person-years/ or number of total participants plus follow-up period, we will estimate the missing information as previously described [27, 28].
3. Non-linear dose-response relation: in addition, we will explore whether there is indication for a non-linear dose-response relation between dietary factors and chronic diseases. We will perform cubic spline regression models and evaluate non-linearity by using a likelihood ratio test [29].

To explore heterogeneity between studies, we will use the Q test and the ${I}^2$ statistic (with a value of ${I}^2>50\%$ considered to represent potentially important heterogeneity [30]). In addition, to identify potential sources of heterogeneity, we will stratify the meta-analysis by subgroups (age, length of follow-up, dietary assessment method) and use meta-regression analysis.

#### Assessment of the transitivity assumption

Transitivity is the fundamental assumption of indirect comparisons and network meta-analysis, and its violation threatens the validity of the findings obtained from a network of studies. Our inference about the plausibility of transitivity will be based on the following criteria:

- We will assess the similarity and comparability of each dietary factor as well as for the control/reference groups when they appear in different studies. For example, we will assess whether discrepancies in amount of intake or definition of exposure of the same foods across studies are likely to render some nodes in the network intransitive.
- We will assess whether the potential effect modifiers (see the *Data Extraction* section) are similarly distributed across the available direct comparisons in the network.

#### Network meta-analysis

If the identified studies appear to be sufficiently similar with respect to the effect modifiers and transitivity is likely be plausible, we will perform a random effects network meta-analysis for each outcome to estimate all possible pairwise relative effects and obtain a clinically meaningful relative ranking of the different food groups. We will use contrast-level data (HRs, RRs or ORs separately) as we do not expect arm-level data to be available. We will account for the correlation in studies with multiple dietary groups when the required data are available to obtain the covariance of the effect sizes from such studies. If we will not be able to obtain these covariances, we will treat the effect sizes from these studies as independent and we will assume different correlation values in a sensitivity analysis. We will present summary HRs, RRs or ORs in a league table. We will also estimate the relative ranking of the different food groups for each outcome using the distribution of the ranking probabilities and the surface under the cumulative ranking curves (SUCRA) [31]. For each outcome, we will assume a common network-specific heterogeneity parameter and we will estimate the predictive intervals to assess how much this heterogeneity affects the relative effects with respect to the additional uncertainty anticipated in future studies [32].

#### Assessment of inconsistency

To evaluate the presence of statistical inconsistency (i.e. disagreement between the different sources of evidence) in the data we will employ both local and global approaches [33]. Specifically, we will use the loop-specific approach [34] to detect loops of evidence that might present important inconsistency as well as the node-splitting approach [35] to detect comparisons for which direct estimates disagree with indirect evidence from the entire network. Global methods investigate the presence of inconsistency jointly from all possible sources in the network. For this purpose, we will use the design-by-treatment interaction model and the ${I}^2$ statistic [36, 37].

#### Exploration of heterogeneity and inconsistency

In the presence of important heterogeneity and/or inconsistency, we will explore the possible sources. If sufficient data will be available, we will run network meta-regression analyses to account for differences by duration of follow-up of the studies, sex, dietary assessment method, level of physical activity, body mass index, smoking status and outcome relevant baseline risk factors (e.g. blood pressure, dyslipidemia).

#### Small-study effects and publication bias

We will use the comparison-adjusted funnel plot [23] to assess the presence of small-study effects in the network and contour-enhanced funnel plots [38] to investigate whether funnel plot asymmetry is likely to be explained by publication bias. We will also run network meta-regression models that account for differences in the relative effects between smaller and larger studies [39].

#### Sensitivity analyses

We will assess the sensitivity of results for the primary outcome by analysing only studies considered being at low risk of bias.

#### Implementation and software

We will fit all analyses described in a frequentist framework using Stata [40] (*network* package [41]), and we will produce presentation tools with the *network graphs* package [42].

### Quality of the evidence

We will first use our recently developed NutriGrade-tool to evaluate and judge the meta-evidence for pairwise comparisons, which has been especially developed for nutrition research to address specific requirements for this research field [21]. Then, to infer about the quality of evidence from the network meta-analysis will combine our judgment about the direct comparisons with their contributions in the estimation within the network as described in Salanti et al. [33].

## Discussion

This systematic review and network meta-analysis will be the first to summarize and compare the effects of different foods or food groups on all-cause mortality, cardiovascular disease, type 2 diabetes and cancer, using both direct and indirect evidence. This analysis will show which foods or food groups, if any, might be the most promising in the prevention of chronic diseases. Our systematic review will be of great value to national and international authorities for evidence-based nutritional recommendation/guidelines, regarding the prevention of chronic diseases. Moreover, our results can be implemented to develop new diet quality indices/scores.

## Additional file

Additional file 1: PRISMA-P checklist. (DOC 82 kb)
